# They also serve who stand and wait…..

**DOI:** 10.4103/0019-5545.37659

**Published:** 2007

**Authors:** G. Swaminath

**Affiliations:** Department of Psychiatry, Dr. B. R. Ambedkar Medical College, Kadugondanahalli, Bangalore - 560045, India

My predecessor, Dr. T S S Rao, taking over as Editor of the Indian Journal of Psychiatry (IJP), began[1] by quoting John F Kennedy “Change is the law of life……” Little did he or any one of us realize then how ironically prophetic that quote would be. The editorial board of the IJP was propelled into transformation by circumstances. The schedule of the publication of our journal was pushed into disarray. This turmoil enforced a period of a few months when the journal seemingly went into slumber. Bringing it back to the pace required to produce a quality output entailed the expenditure of great time and effort and the editorial team had to work frenetically to succeed in bringing out the last two issues in a time span of two months.

It is fashionable to keep patting the editorial team on the back, for a job voluntarily chosen. In the process, the actual contributors to the quality of the journal, mainly the triad of authors, reviewers and the publishers are rarely acknowledged.

Authors have chosen the IJP as a platform to disseminate the results of their research. Others have shared with us their wisdom or their vast knowledge. Some have shown us their literary creativity. Their involvement requires our gratitude. They have chosen to link their accomplishments with the journal and they should be complimented.

The reviewers do a thankless job of reviewing manuscripts behind the scenes. Their contributions are usually taken for granted. Their output, usually invisible, ensures the quality of the final product. It is, however, a humbling experience to witness the passion which many reviewers put into their work, the meticulousness with which they go through every line and sometimes comment on (and even painstakingly correct at times) the grammar and construction. I have had occasion to go through the comments of one overseas reviewer, a researcher on the topic of the submission, who literally rewrote the English of the manuscript, as he opined that the article, which was from another culture with deficient psychiatric infrastructure, had merit but would not pass through due to poor language. Good reviewers are often researchers themselves and do empathise with those who have done work in other centres contributing to the knowledge bank, but are unable to publish due to lack of skills in writing and publishing papers. The unsung and anonymous group of reviewers requires our sincere salutation.

The third arm of the triad, the publishers, bring out the final product. Their job involves coordinating with the various departments from manuscript submission to publishing and printing of the journal. The present publishers have been innovators and entrepreneurs, led by a medically qualified person and have been publishing many journals. Their online manuscript submission system and electronic peer review system has stood us in good stead and helped us weather the uncertainties this year by expediting peer review, editorial and production work as well as distribution. Due to this system we have been able to reach out to many foreign reviewers. Our published articles have been available for electronic viewing online at www.indianjpsychiatry.org via open access thanks to the publishers.

This is probably my last issue as editor of this journal and I dedicate this issue to the committed triad of author, reviewer and publisher for making IJP a product to be proud of.

I am grateful to the membership of IPS, the President, the Secretary and other office bearers of the society, who had confidence to ask me to become interim editor. I am indebted to the Hon Chief Advisor and other editors who have helped me and the journal in this difficult phase. My thanks to members of the Editorial Board, Journal Committee, National and International Advisory Boards, statistical consultants and many others who have been of great help to me. Working earlier in the capacity of Assistant Editor and later as the Editor, has been a valuable, enriching and memorable experience which I will cherish always.

My best wishes to the IJP which is reaching its Golden Jubilee this year.
